# Distinguishing between Similar Miniproteins with Single-Molecule Nanopore Sensing: A Computational Study

**DOI:** 10.1021/acsnanoscienceau.1c00022

**Published:** 2021-12-28

**Authors:** Sebastian Cardoch, Nicusor Timneanu, Carl Caleman, Ralph H. Scheicher

**Affiliations:** † Department of Physics and Astronomy, Uppsala University, Box 516, SE-751 20 Uppsala, Sweden; ‡ Center for Free-Electron Laser Science, Deutsches Elektronen-Synchrotron DESY, Notkestraße 85, 22607 Hamburg, Germany

**Keywords:** nanopores, single-protein sensing, miniprotein, molecular dynamic simulations, density functional theory, relative surface accessibility, dwell time

## Abstract

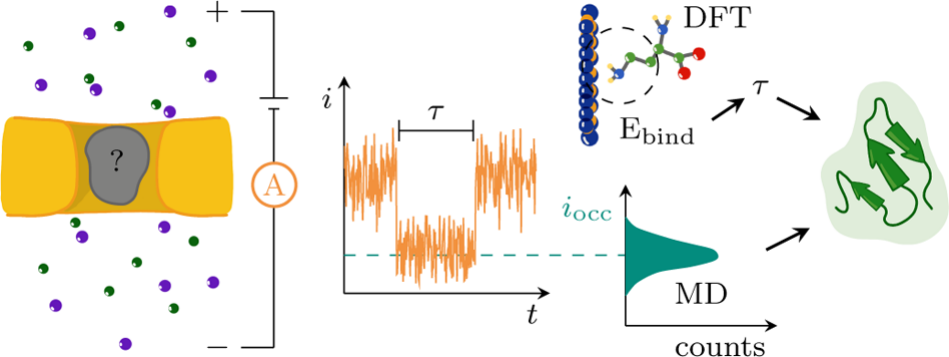

A nanopore is a tool in single-molecule sensing biotechnology that offers label-free identification with high throughput. Nanopores have been successfully applied to sequence DNA and show potential in the study of proteins. Nevertheless, the task remains challenging due to the large variability in size, charges, and folds of proteins. Miniproteins have a small number of residues, limited secondary structure, and stable tertiary structure, which can offer a systematic way to reduce complexity. In this computational work, we theoretically evaluated sensing two miniproteins found in the human body using a silicon nitride nanopore. We employed molecular dynamics methods to compute occupied-pore ionic current magnitudes and electronic structure calculations to obtain interaction strengths between pore wall and miniprotein. From the interaction strength, we derived dwell times using a mix of combinatorics and numerical solutions. This latter approach circumvents typical computational demands needed to simulate translocation events using molecular dynamics. We focused on two miniproteins potentially difficult to distinguish owing to their isotropic geometry, similar number of residues, and overall comparable structure. We found that the occupied-pore current magnitudes not to vary significantly, but their dwell times differ by 1 order of magnitude. Together, these results suggest a successful identification protocol for similar miniproteins.

## Introduction

In single-cell analysis, variations in the protein content between cells enable the study of cell diversity in stem cells or tumor cell growth.^1^ In both applications, a single-cell resolution is necessary to reduce biological noise.^1^ Challenges in this field are stochastic protein expression within the cell and the lack of protein amplification methods similar to those available for nucleic acids.^1,2^ To achieve genuine single-cell resolution, protein-sensing techniques with efficient sample manipulation and sensitive detection are required.^1,2^

Solid-state nanopores have been proposed as a single-molecule sensing tool^3−6^ that offers potential in single-cell analysis. In a typical setup, a membrane separates two chambers with electrolyte solution connected by a nanosize opening. A voltage applied across the membrane generates a steady ionic current. A charged analyte of interest placed in one of the chambers translocates through the nanopore by an interplay between electrophoresis, electroosmosis, Brownian motion, and surface interactions with the membrane. When the analyte occupies a volume in the pore region, the flow of ions is modified and becomes sensitive to the analyte’s size, shape, and surface properties.^7^ These ionic current modulations measured over time can be used to identify biomolecules.

Due to their advantage as a label-free method, nanopores have been employed to study ions, viruses, DNA, and RNA.^8,9^ Nanopores have been successfully employed for DNA sequencing, and recent advancements in the field have focused on the detection and identification of proteins.^3,7,9−17^ However, protein sensing presents an increased level of difficulty compared to DNA sequencing in several aspects. Proteins fold in complex three-dimensional structures, their net charge can vary significantly, and they generally exhibit a nonuniform charge distribution.^15^ Additionally, residue modifications can yield distinct functional forms of the proteins^12^ that are likely present in smaller concentrations.^18^ Some of these attributes have been shown to have an effect on the direction and mechanism for translocation.^19^ A potential solution to overcome this complexity is to examine smaller systems, for example, miniproteins or domains that remain stable outside of the larger protein structure.

Miniproteins are proteins with less than or equal to 40 amino acids with secondary and tertiary structures^20^ that stay in their native form as a result of a small hydrophobic core, with the help of cross-link atoms or through chemical stability.^21^ Miniproteins have a limited number of secondary structures and are suitable candidates to study folds in larger conformations,^20,22^ aid in drug development,^21^ and address the folding problem.^23^

The mechanism to study miniproteins using nanopores can be split into two stages: (1) the protein’s diffusion toward the pore entrance and (2) its passage through the pore.^15,24^ Information about the structure and conformation of the protein in the latter stage is described by both the magnitude and the duration of the ionic current modulation.^25^ Small pore diameters are desirable for this task, because relative variations in the magnitude of the ionic current decrease as the pore diameter grows.^26^ For pore sizes comparable to the size of the protein, the biomolecule must overcome an activation energy to be captured by the pore.^15,27,28^

Computationally, the method of molecular dynamics (MD) can give an atomic-level description of the transport of proteins through the nanopore.^25^ MD simulations offer a high level of accuracy by treating all atoms in the solvent, pore, and solution explicitly.^8^ This approach comes at a computational cost that limits time scales accessible to MD, where typical translocation times for proteins and DNA range from microseconds to milliseconds.^25^ To accelerate this process, several methods have been explored, for example, large transmembrane voltage, steered molecular dynamics,^25^ harmonic restraints,^29^ external forces to keep the molecule fixed along the pore axis,^30^ or omitting binding interactions between the pore and analyte.^31^ Many of these methods distort the conformational structure of the protein^29^ and are limited by the stability of the protein under these conditions.^25^ Dwell times are prescribed by the acceleration method of choice. We therefore identify a need to obtain theoretical predictions of the dwell time of the analyte.

**Figure 1:**
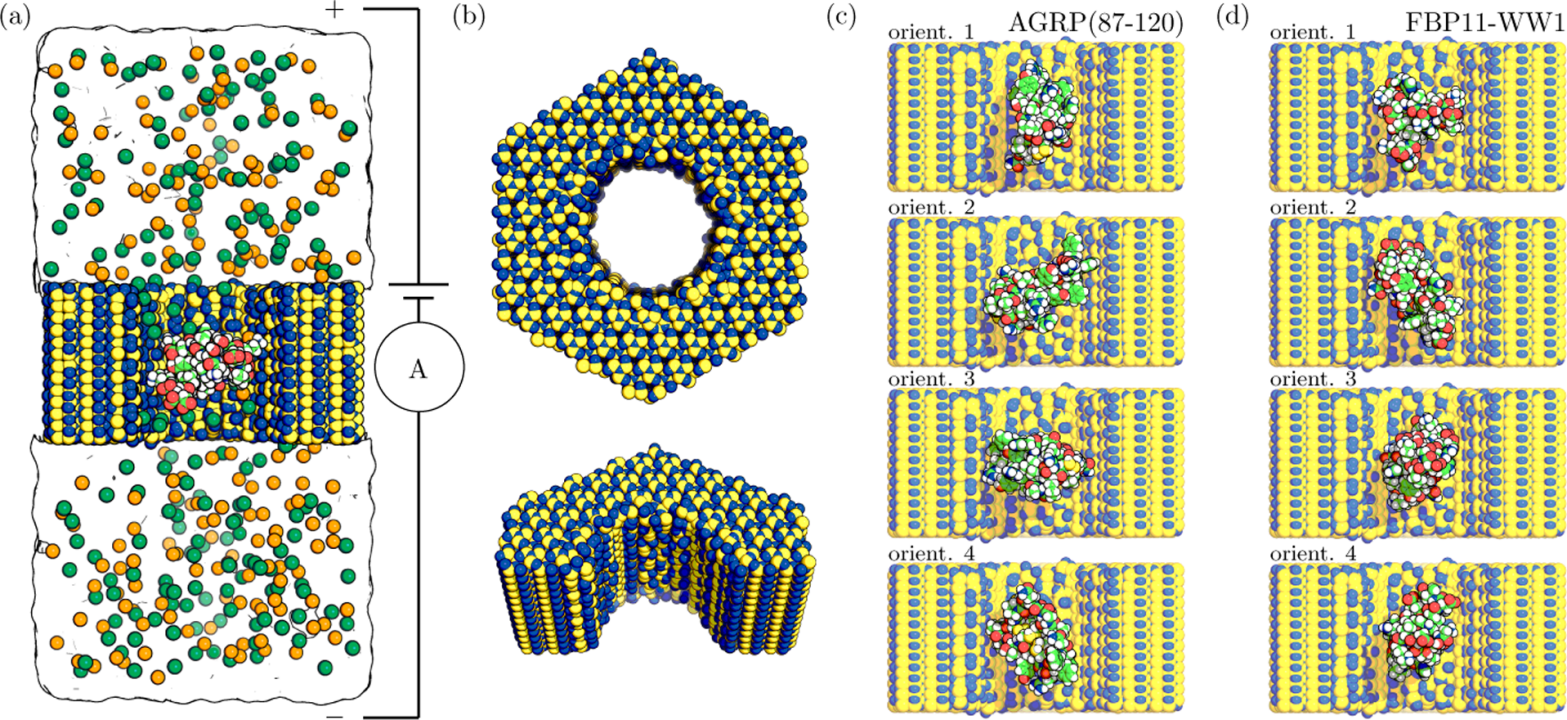
(a) All-atom model of the nanopore and miniprotein system surrounded by water (boundary outlined by a solid line) and a 1 M concentration of potassium (dark-green) chloride (orange) ions. (b) The silicon (yellow) nitride (blue) membrane is hexagonal in shape with 38 Å thickness, and the pore is cylindrical-shaped with a 35 Å pore diameter. (c,d) Three starting orientations for miniproteins AGRP(87-120) and FBP11-WW1 explored in MD simulations.

In this work, we explore the sensing abilities of a nanopore on two miniproteins in their folded state. We assessed the identification of these biomolecules from a combination of occupied-pore currents based on MD simulations and dwell time estimates based on electronic structure calculations. The occupied-pore currents were determined by creating an all-atom model, placing the miniproteins at the center of a pore in four different initial orientations, as shown in Figure 1. The simulations were carried out without restraint on the proteins and using voltage magnitudes accessible to experiments. To determine dwell times, we computed the total energy between pore wall and individual amino acids over many separation distances. We employed relative surface accessibility (RSA) values as weights to derive the interaction energy of the miniproteins and combined these with numerical solutions to the trajectory of miniproteins in the absence of surface contacts.

We selected two roughly isotropic domains from larger proteins found in human cells with a similar number of residues that form comparable secondary structures. Disregarding the limits on temporal resolution, we believe these proteins could be challenging to distinguish with a nanopore because of these shared similarities. AGRP(87-120) (3.9 kDa) is a modified C-terminal domain of the regulatory agouti-related protein that folds into two beta-strands and remains stable by the presence of three cysteine cross-links.^32^ FBP11-WW1 is the first WW domain (3.6 kDa) of splicing enhancer protein FBP11, which folds into a beta-sheet and possesses a small hydrophobic core.^33^

## Results and Discussion

MD simulations of ionic current inside a pore occupied by either AGRP(87-120) or FBP11-WW1 in their native state reveal the identification of these two miniproteins is not possible based on current magnitudes. DFT simulations of the interaction energies between the pore-wall and amino acids that make up AGRP(87-120) and FBP11-WW1 yield translocation-times that differ by 1 order of magnitude. Put together, MD and DFT results provide enough information to predict the successful identification of these miniproteins in ideal experimental conditions.

**Figure 2:**
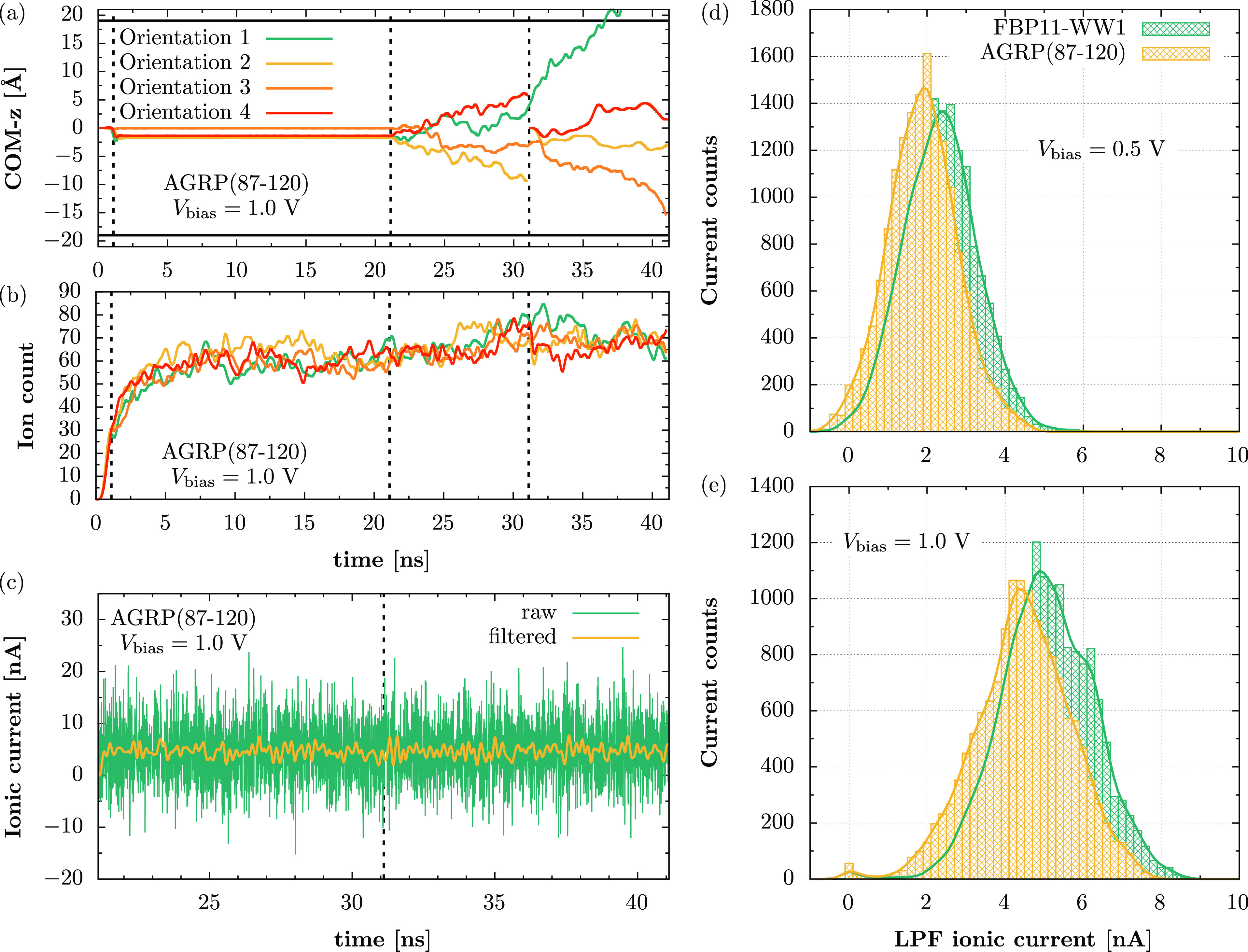
(a) *Z*-component for the center of mass (COM) of the miniprotein. Horizontal lines at ±19 Å represent the thickness of the membrane, and vertical dashed lines represent changes in the simulated environment, as described in the text. (b) The instantaneous number of ions inside the pore region. (a,b) Results are smoothed out using a LPF with 1 GHz cutoff frequency. (c) Raw and LPFd occupied-pore currents for the last 20 ns computed based on ion trajectories. (d,e) Histograms comparing LPFd occupied-pore ion currents of both miniproteins for all three orientations.

To compute the occupied-pore ion currents, we ran all-atom simulations with one miniprotein placed at the center of the pore subject to 0.5 and 1.0 V bias voltage, respectively. After a period of minimization and equilibration, we turned on the bias voltage and applied harmonic restraints on all atoms to stop the solvent from leaving the pore region. The miniprotein’s center of mass and the instantaneous number of ions inside the pore were monitored and the results are summarized in Figure 2a,b for AGRP(87-120) at 1.0 V bias voltage. Analogous data at 0.5 V and for FBP11-WW1 are presented in the Supporting Information. We observed the instantaneous number of ions reached a steady-state after 10 ns that could resemble experimental conditions. We continued to evolve this system for a further 20 ns in steps of 10 ns, with restraints on the miniprotein now removed. We regarded these conditions to be more in line with experiments since the miniprotein was free to respond to the applied electric field, interact with the membrane, and escape the pore. We were only interested in the occupied-pore signal. In cases when the miniprotein moved far away from the center of the pore, the simulation was restarted using an earlier time step. Such restarts show up as discontinuities in the position of the miniprotein.

Based on ion trajectories captured from the last 20 ns, we computed the ionic current according to eq 2, with results for AGRP(87-120) over a single orientation shown in Figure 2c. We reduced the noise in the computed ionic current by applying a low pass filter (LPF) with a cutoff frequency of 5 GHz. We repeated these steps for the remaining orientations of AGRP(87-120) and FBP11-WW1 and simulations subject to a 0.5 V voltage bias. These results are included in the Supporting Information. In Figure 2d,e, we employed histograms to compare the occupied-pore currents. We found some variability in the distributions at both bias voltages but not significant enough to be able to uniquely distinguish these proteins. We attribute similarities in the histograms to the roughly isotropic shape of the proteins. Furthermore, we monitored the miniproteins’ dipole orientation and generated ionic current histograms when the dipole aligned or did not align with the external field (details in the Supporting Information). We found in general the alignment of the proteins did not alter the ionic current distributions. In most simulations, the miniproteins remained in a folded state, except for two occasions at 1.0 V (orientation 1 after 35.5 ns and orientation 3 after 37.5 ns) where AGRP(87-120) left the pore or became partially unfolded due to its interaction with the pore walls and external field. Ionic current values at these times were excluded from the histogram.

**Figure 3:**
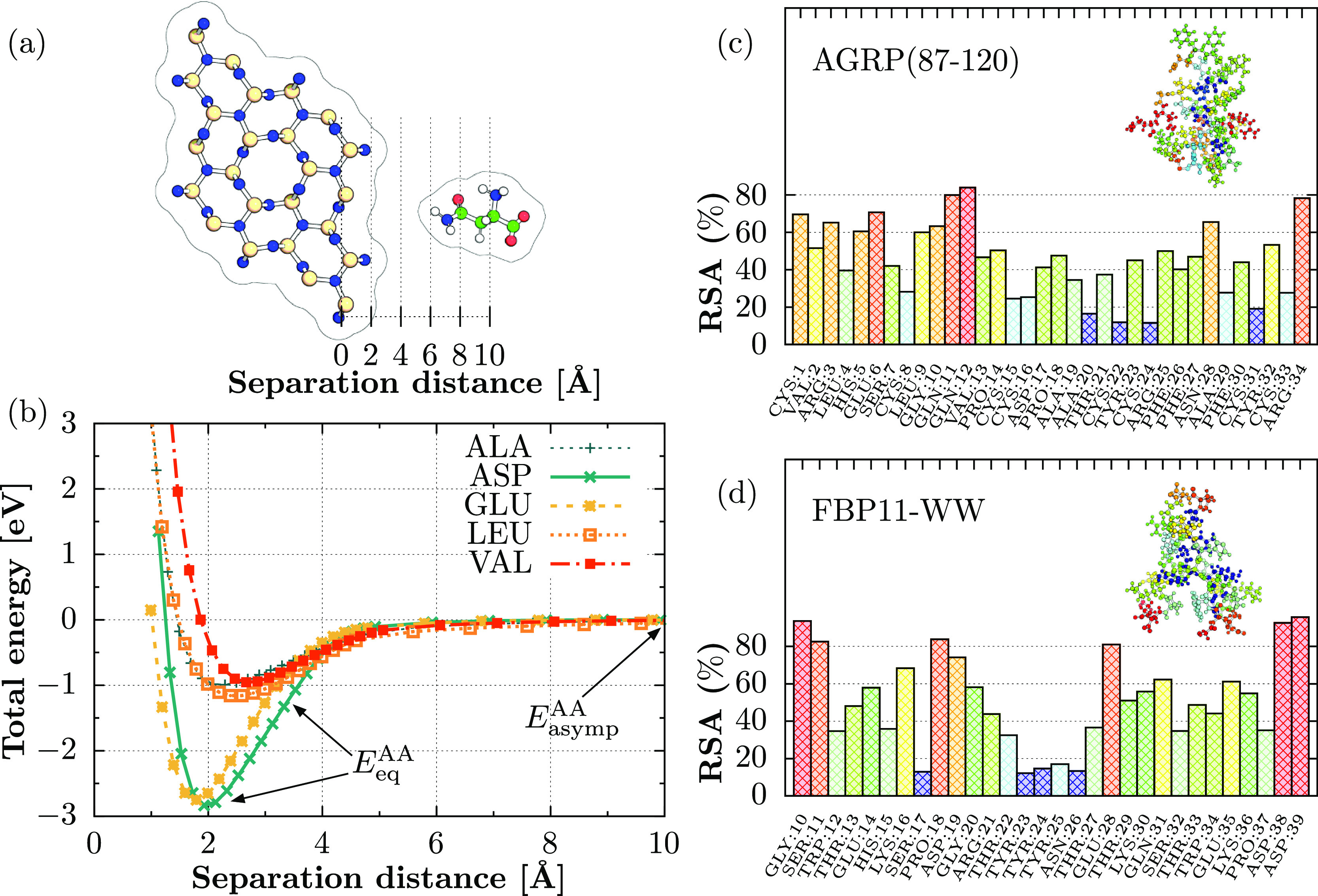
(a) Top-view of the all-atom model, where a silicon nitride slab mimics the pore-wall from the MD model and where amino acid ASN is oriented with its R-group facing the surface. (b) The total energy of the amino-acid-pore-wall system as a function of their separation distance for selected amino acids. (c,d) RSA values for both miniproteins colored with a rainbow spectrum where red represents the most and blue the least accessible amino acid. These values were determined based on the miniproteins’ sequence using the local structural predictive tool NetSurfP-2.0 (ref (34)).

To investigate the translocation time, we consider a model where the dwell time is proportional to the transition between two states: when the miniprotein is close to the pore-wall at a separation distance *s*_1_ with energy *E*_eq_ and when it is far away at *s*_2_ with energy *E*_asymp_. The Boltzmann distribution describes the probability of being in a single state. The transition *s*_1_ → *s*_2_ is given by the ratio of these probabilities such that the translocation time τ becomes
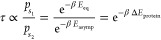
1where Δ*E*_protein_ is the interaction energy between the miniprotein and pore-wall and β^–1^ is the characteristic energy of the transition. For a 1 V bias voltage, we reasoned β^–1^ to be in the order of 1 eV for AGRP(87-120) given that the charge of this miniprotein is +1e and 2 eV for FBP11-WW1 given that the charge for this miniprotein is −2e. We computed the interaction energy for each amino acid in the miniproteins’ sequence from electronic structure calculations. For this, we employed an atomic model like the one shown in Figure 3a. The total energy calculations of the amino acid (AA) and pore-wall computed over many separation distances provided the energy at the equilibrium distance *E*_eq_^AA^ and at the asymptotic limit *E*_asymp_^AA^. From their difference, we obtained the interaction energy of each amino acid Δ*E*^AA^. Figure 3b shows the total energy results for selected amino acids at various separation distances, and a complete summary of interaction energies can be found in Table 1. All amino acids showed attraction to the pore-wall, which is understandable based on the dielectric and polar nature of silicon nitride and amino acids, respectively. The amino acid is uncharged but has a dipole moment that induces a polarization in the silicon nitride. In a typical experimental setting, the system is subject to an external electric field that influences the membrane’s polarization. We omitted this effect in the electronic structure calculations.

**Table 1:** Summary of Amino Acid Interaction Energies Based on the Energy Difference at the Equilibrium Point Determined Using Quadratic Spline Interpolation and Asymptotic Limit Determined by a Morse Potential Fit^a^

R-group	amino acid	– Δ*E*^AA^ [eV]
positively charged	HIS	0.8913
	ARG	2.4674
	LYS	2.9291
		
negatively charged	ASP	2.8251
	GLU	2.7363
		
uncharged polar	ASN^b^	1.2610
	GLN	0.9411
	SER	1.4538
	THR	0.9887
	TYR	1.2834
		
nonpolar	ALA	0.9876
	VAL	0.9424
	LEU	1.1341
	PRO	1.0948
	PHE	0.8881
	TRP	1.0852
	GLY	1.4880
	CYS	1.4944

a Amino acids are organized by the charge of their side chain.

b At equilibrium, we used the lowest computed value instead of the quadratic interpolation.

The connection between the interaction energy of the miniprotein and that of individual amino acids was made via a selection model. We proposed that at most four amino acids sitting at the equilibrium distance from the pore wall and had a contribution on Δ*E*_protein_. The first amino acid was selected through a weighted random choice using relative surface accessibility (RSA) values, summarized in Figure 3c,d, as weights. The remaining three were chosen at random from a list of the geometrically closest five amino acids to the initially selected amino acid. To compute Δ*E*_protein_, we assumed linearity, meaning the sum of energies from each amino acid gave the interaction energy of the miniprotein. This four-amino-acid selection process was repeated 500 000 times for each miniprotein, which gave rise to a gamma distribution with a mean and standard deviation
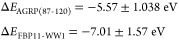


We carried out a sensitivity analysis for the choices made to obtain these energies, which is discussed in the Methods section. To arrive at dwell times, we assumed the interaction between the pore wall and solvent was a leading contributor to the permeation. This is only realized if the pore size is comparable to the size of the translocating molecule. We disregarded any influence from entropy since the miniprotein remained in a folded state inside and outside the nanopore. We also omitted conformation restrictions or deformation due to surface contacts and excluded the influence from the electroosmotic and electrophoretic flow. We first considered a noninteracting miniprotein Δ*E*_protein_ ≈ 0 has a characteristic dwell time τ = *A* e^–β·(0)^ = τ_0_, where *A* is some proportionality constant unique to each protein. In the case where the interaction between the solvent and the pore-wall is repulsive, Δ*E*_protein_ > 0 and thus τ < τ_0_. In the case where interaction is attractive, Δ*E*_protein_ < 0 and thus τ > τ_0_. To determine *A*, we reasoned the miniprotein of charge *q* and mass *m* placed at the entrance of the pore is subject to an electrostatic force and a drag force. Guided by the COM results from MD simulations, we estimated the velocity of the miniprotein to be small  (0.1 m/s) and the drag term could be taken to be proportional to the velocity. This approximation neglected the contribution from water molecules and ions on the relative movement. We derived, based on these forces, an expression for the velocity and position of the miniproteins as a function of time and numerically solved for A (details in the Supporting Information). We found the noninteracting dwell times for both miniproteins to be on the order of nanoseconds. When we factored in the interaction with the pore walls, based on the energies calculated for each miniprotein, we found the following dwell times ranges at 1.0 V bias voltage
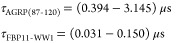


Looking at these results, we first noticed some contradictions in the dwell time obtained from DFT calculations and the COM movement of the proteins computed from MD simulations. In the 1.0 V case, the COM’s time evolution shows FBP11-WW1 is displaced less than AGRP(87-120). Such an observation suggests longer translocation times, opposite to what the dwell time model predicts. We identified five factors that could explain this discrepancy: (1) the number of MD simulations is not enough to sample all possible orientations that the protein can have inside the pore. The COM motions for the three starting orientations follow translocation pathways but do not provide a complete description of the genuine distribution of the translocation times of these proteins. (2) In experiments water molecules partially screen the miniprotein’s charge. The TIP3P water model used in MD simulations is nonpolarizable, and for this reason, the nonbonded interactions between the pore’s surface and solvent in MD simulations are overestimated. (3) The force fields employed on the protein and silicon nitride are not parametrized to accurately describe their interaction. Using an appropriate exchange-correlation functional, DFT is designed to describe the van der Waals interactions between each amino acid and membrane to a high degree of accuracy.^35^ We rely on the method we developed based on electronic structure calculations to give us a better account of the dwell time. (4) We also made approximations in the electronic calculations that could negatively impact the accuracy of the results. The system lacked water and ions, and we considered a single conformation and orientation of the solvent with respect to the pore, ignored the influence of neighboring amino acids, and omitted the presence of an external field. The absence of water introduces an approximately systematic error in the total energy of the system at each separation distance between the pore wall and the amino acid. (5) The influence from the electrophoretic and electroosmotic flows of ions and water were not included in our model. This effect is noticeable in the different trajectories taken by the same miniprotein during MD simulations, while in the translocation model we assume the miniprotein translocates along the direction of the electric field.

## Conclusions and Outlook

In this paper, we set out to investigate if a thin silicon nitride nanopore could be employed to distinguish two roughly isotropic miniproteins with similar residue length and secondary structure. Our evaluation combined ionic currents of the occupied pore and translocation times for each miniprotein. We found that although the occupied-pore ionic currents for both miniproteins were similar, their dwell time differed by 1 order of magnitude. The combined results predict AGRP(87-120) and FBP11-WW1 can be uniquely identified in experiments.

To obtain these results, we presented a method to estimate dwell times based on DFT that circumvents the computational demands required to simulate the entire permeation of the protein. The interaction between the pore wall and at most 20 amino acids is enough, and the computational resources do not need to scale up with the size of the protein. A disadvantage of this approach is that it cannot reveal the mechanism for permeation, which is relevant since different events can take place near or inside the pore that complicates the translocation picture.^24^

In experimental settings, sensing small proteins is challenging due to the low signal-to-noise ratio and generally fast translocation times.^15,36^ Detection limits are set by the temporal resolution of the amplifier and noise reduction techniques utilized.^15^ Sensing can be improved by increasing the bandwidth or slowing down the protein’s permeation.^15^ With this technical limitation in mind, direct comparison with experiments can only be made possible by studying larger proteins, which we see as a natural follow-up to our investigation.

Future studies can further develop the model used to derive the comparative dwell times. The interaction energy for the two miniproteins disregards thermal fluctuations currently, and this contribution could be added as a source of noise in the energy distribution. The inclusion of water, ions, sampling different amino acid configurations, and external field in the electronic structure computations would give a higher level of accuracy on the pore wall and amino acid interaction. Time evolutions for RSA values and contact points between analyte and pore can be obtained directly from MD simulation trajectories. Such an approach can improve the selection scheme to calculate the interaction energy of the entire protein. By combining MD trajectories, we can potentially evaluate dwell times for different protein conformations and unfolded states.

## Methods

### Molecular Mechanics Methods

To built the all-atom model and carry out MD simulations, we based our work on the methods presented in Aksimentiev et al.,^37^ Aksimentiev and Schulten,^38^ and Comer et al.^39^ We employed the molecular dynamics simulation package NAMD^40^ coupled with the CHARMM36 force field^41^ to describe miniproteins TIP3P water molecules and ions and CHARMM adaptation of the MSXX force field^37,42^ to model the silicon nitride membrane. We used the SETTLE algorithm to fix hydrogen bonds in water and the particle mesh Ewald (PME) algorithm to compute long-range electrostatics with a grid size ≤1 Å removing drift momentum before each calculation. We used a multiple-time-step integration method to compute bonded forces every 1 fs, short-range nonbonded forces every 2 fs, and long-range electrostatics every 4 fs. We excluded 1–3 bonded pairs from nonbonded interactions and used a 10–12 Å smooth switching function for van der Waals interaction.

Each all-atom model was first minimized for 0.01 ns and equilibrated for 1.0 ns under NPT conditions. Simulations in the presence of bias voltages were carried out under NVT conditions. The simulation-cell area, along the membrane’s plane, was fixed from the last equilibration step. The length normal to the plane was taken as the average from the last 0.5 ns and atom positions were scaled accordingly. Finally, we employed a spring constant of 1.0 kcal/(mol Å^2^) to harmonically restrain all atoms in the miniprotein.

For NVT and NPT simulations, the temperature was fixed at 298 K using a Lowe–Andersen thermostat^43^ with a cutoff radius of 3.3 Å and a collision rate of 50 ps^–1^. We found it appropriate to employ this thermostat in the simulations since it conserves momentum.^43,44^ NPT simulations were performed using a Nose–Hoover Langevin piston with a target pressure of 1.0 atm, a piston period of 200 fs, and piston decay of 100 fs. To maintain pressure, we defined a flexible cell but kept the ratio along the membrane plane constant. Finally, we captured all atoms’ positions every 10 ps during equilibration and every 5 ps for the remaining simulations.

#### All-Atom Model

We built a cylindrical-shaped nanopore of diameter *d* = 35 Å on a hexagonal silicon nitride membrane with side length *s* = 37.98 Å and thickness *h* = 37.73 Å. The charges of silicon and nitrogen atoms were adjusted by ≈0.07% to keep the membrane neutral and account for the unequal loss of atoms after creating the pore. The ratio of silicon and nitrogen atoms at the pore surface depends on the chosen pore diameter. We found, inspired by the procedure outlined in ref (37), the surface could be made more homogeneous by minimizing and equilibrating the membrane in vacuum. For more details, see the Supporting Information. We obtained a pore surface with an improved silicon and nitrogen ratio of 0.741 compared to the initial ratio of 0.694.

The nanopore was occupied by miniproteins AGRP(87-120) or FBP11-WW1 at four different starting orientations with respect to the pore axis. The system was solvated with TIP3P water molecules up to a height of 80 Å and ionized with a 1 M concentration of potassium chloride using VMD’s^45^ plugin *solvate* and *autoionize*, respectively. Based on the protonation state of the titratable side chain at physiological pH 7.1 and using the open-source web server H++ 3.0,^46−48^ we found the net charges of AGRP(87-120) to be +1e and FBP11-WW1 −2e. AMBER-style files were transcribed to CHARMM-style using the MMTSB tool set.^49^ We added excess ions to make the all-atom system neutral.

#### Silicon Nitride Permittivity

Silicon nitride is a dielectric that polarizes under the influence of an external field, with the amount of polarization described by its relative permittivity ϵ_r_. The bonded parameters of the MSXX force field give a relative permittivity close to vacuum, but based on methods described in refs (39 and 50), we arrived at its experimentally known value of 7.5 (ref (51)) by applying a harmonic restraint of 7.0 kcal/(mol Å^2^) to all atoms. We built a silicon nitride sphere with a volume similar to the hexagonal membrane and carried out simulations, systematically adjusting the harmonic restraint. We later computed the relative permittivity based on changes to the electric field inside the sphere with and without the influence of a uniform external field induced by a 1.0 V bias voltage. We derived changes in the electric field inside the sphere from time-averaged dipole moment changes and found a logarithmic dependence between ϵ_r_ and the harmonic restraint. We also found ϵ_r_ remained unchanged at different bias voltages, but high uncertainty at lower voltages meant we could not identify a clear trend. For results, see the Supporting Information.

#### Ion Conductivity

The diffusivity of the TIP3P water model is large compared to experimental values.^52^ We made systematic adjustments to the Lowe–Andersen thermostat radius *R*_T_ to introduce an artificial viscosity, modify the water diffusivity, and ultimately obtain a potassium chloride ion conductivity κ_KCl_ close to its experimental bulk value of 11 S/m (refs (38 and 53)). We followed the methods described in ref (38) and computed κ_KCl_ for a cubic cell 4 nm in length, filled with TIP3P water molecules and a 1 M KCl concentration. The conductivity for a *R*_T_ was computed at different bias voltages to obtain κ_KCl_ from the slope of a linear fit. For more details, see the Supporting Information.

#### Ionic Current Calculations

We used the trajectory of ions inside the pore to compute the ionic current according to ref (37).
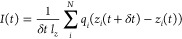
2where *z*_*i*_(*t* + δ*t*) – *z*_*i*_(*t*) is the displacement of the atom *i* along the *z*-direction over a time interval δ*t* = 1 ps, charge of the ion *q*_*i*_ = ±1e, and length of the pore region *l*_*z*_ = 36 Å.

### Density Functional Theory Methods

For electronic structure calculations, we employed the code SIESTA-4.0 (ref (54)) along with the nonlocal van der Waals functional DRSLL^55,56^ to approximate the exchange-correlation term. We carried out all simulations using a double-ζ polarized basis set and a Pulay mixing scheme with a mixing coefficient of 0.02 and a self-consistent cycle history of 5 to reach convergence. The mesh cutoff energy was fixed to 300 Ry according to results from convergence runs. For more details, see the Supporting Information. We selected Abinit’s GGA-PB nonrelativistic pseudopotential for all elements in the system. We fixed the electronic temperature at 300 K with the convergence tolerance for the density matrix fixed at 1 × 10^–4^ eV.

#### All-Atom Model

For the calculations, we modeled the pore surface as a triclinic silicon nitride slab with an approximate thickness of 13 Å, *x*-length 14 Å, and *y*-length 15 Å. Water and ions are omitted from the calculations. A model was built for each amino acid, placed at some arbitrarily chosen but similar position along the *x*–*y* plane. The separation distance between the two objects was defined to be the average position of the two first rows of nitrogen and silicon surface atoms and the lowest *z*-position atom in the amino acid. For small separations, we increased the distance in steps of 0.1 or 0.2 Å and 1.0 Å far away. For comparison, the total energies shown in Figure 3b are normalized such that the asymptotic limit approaches zero.

#### Convergence

To find optimal simulation parameters to calculate the total energy of the system, we executed cell dimension, mesh cutoff energy, and k-point converge simulations. We carried out convergence simulations for the silicon nitride slab, amino acid glutamine, and both objects combined. For results, see the Supporting Information. We checked the influence of mirror images and computed total energies for separation distances along the *z*-direction up to ≈15 Å apart.

### Model Sensitivity Analysis

**Figure 4:**
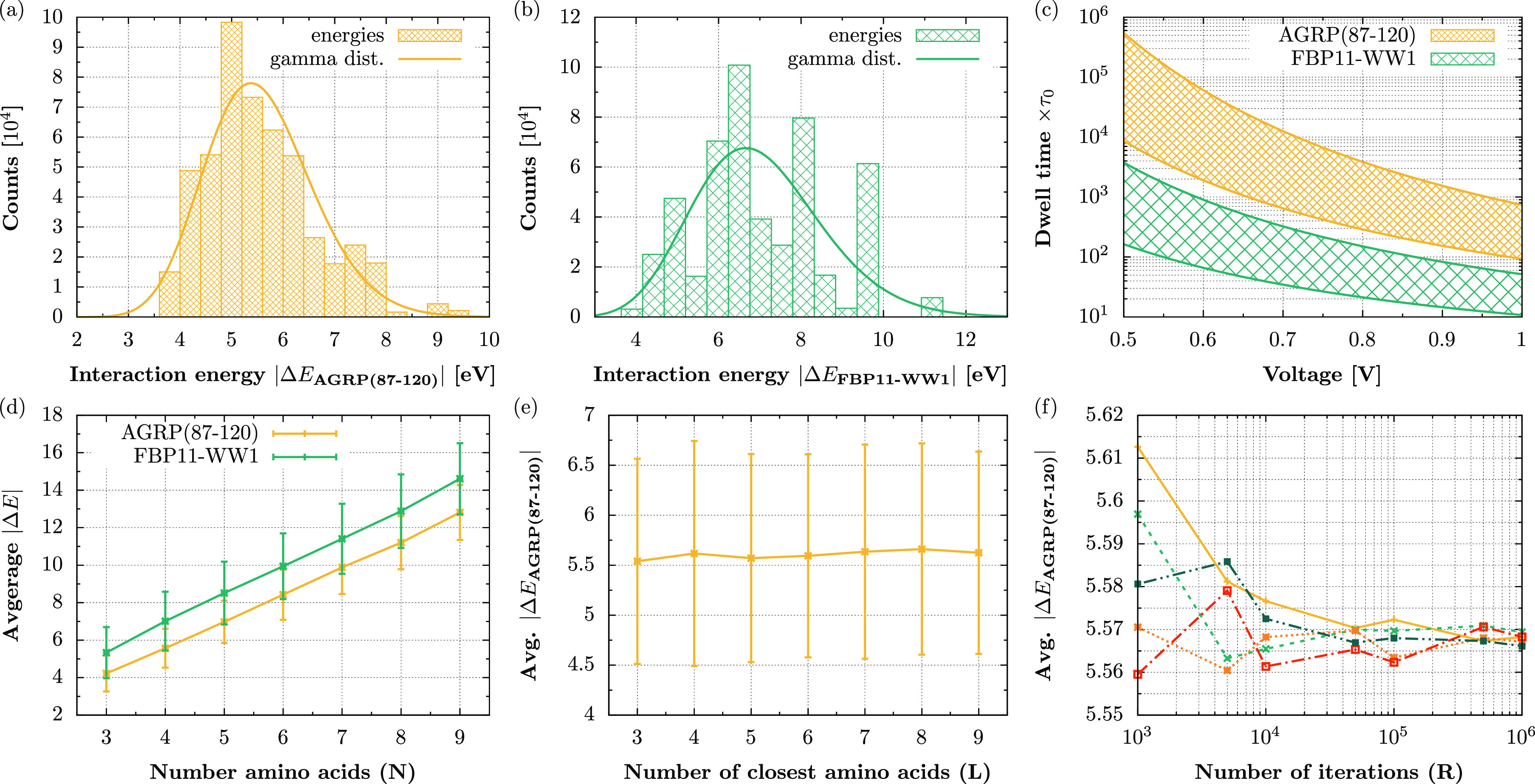
(a,b) Interaction energy histograms for miniproteins AGRP(87-120) and FBP11-WW1 computed 500 000 times give rise to a histogram that is fitted to a gamma distribution to obtain the mean interaction energy and standard deviation. (c) Comparing dwell time of miniproteins based on the mean interaction energies from panels a and b at different bias voltages. (d) Model sensitivity to select *N* amino acids that contribute to the interaction energy. (e) Model sensitivity to select three amino acids from a list of the L-closest amino acids. (f) Model sensitivity to the number of times the interaction energies are computed.

We made certain assumptions in the selection model employed to determine the interaction energies of miniproteins. We explored the sensitivity of our model to changes in these assumptions and show the results in Figure 4. One important parameter is the number of amino acids *N* that contribute to Δ*E*_protein_. In panel e, the interaction energy increases linearly with the inclusion of more amino acids, but the difference in energy between the two miniproteins remains the same. There is a limit in the number of amino acids that can sit at the equilibrium distance from the pore wall, which we assumed to be 4.

## Supporting Information Available

Additional information on the molecular dynamics simulation results, information about the miniprotein, relative permittivity of silicon nitride, pore surface silicon–nitrogen ratio, ionic conductivity, energy convergence, and the terminal velocity of the miniprotein (PDF)
