# Supplementary material for: Distinguishing between Similar Miniproteins with Single-Molecule Nanopore Sensing: A Computational Study
Source: ACS Nanosci Au. 2021 Dec 28;2(2):119–27. doi: 10.1021/acsnanoscienceau.1c00022 (PMC10125149; doi:10.1021/acsnanoscienceau.1c00022)
Supplement: Supplementary file 1 — ng1c00022_si_001.pdf [file ng1c00022_si_001.pdf]

Distinguishing between similar miniproteins with single-molecule nanopore sensing: a computational study

Sebastian Cardoch,[*,†] Nicusor Timneanu,[†] Carl Caleman,[‡,†] and Ralph H. Scheicher[*,†]

†*Department of Physics and Astronomy, Uppsala University, Box 516, SE-751 20, Uppsala, Sweden*

‡*Center for Free-Electron Laser Science, Deutsches Elektronen Synchrotron DESY, Notkestraße 85, 22607 Hamburg, Germany*

E-mail: sebastian.cardoch@physics.uu.se; ralph.scheicher@physics.uu.se

Supporting Information Available

MD simulation results

Additional results for the miniproteins' center of mass and number of ions inside the pore as a function of time are shown in figures 1 and 2, respectively. From the last 20 ns of simulation, we calculated the orientation of the miniproteins' dipole moment from its center of mass. In figure 3 we show the time-evolution of the angle ϕ made between the dipole vector and the +z-axis. We considered the miniprotein aligned with the external field when its dipole moment was at an angle $\leq 50^o$ with the z-axis. Based on the alignment, we generated normalized histograms of the LPF ionic current. These results are shown in figure 4.

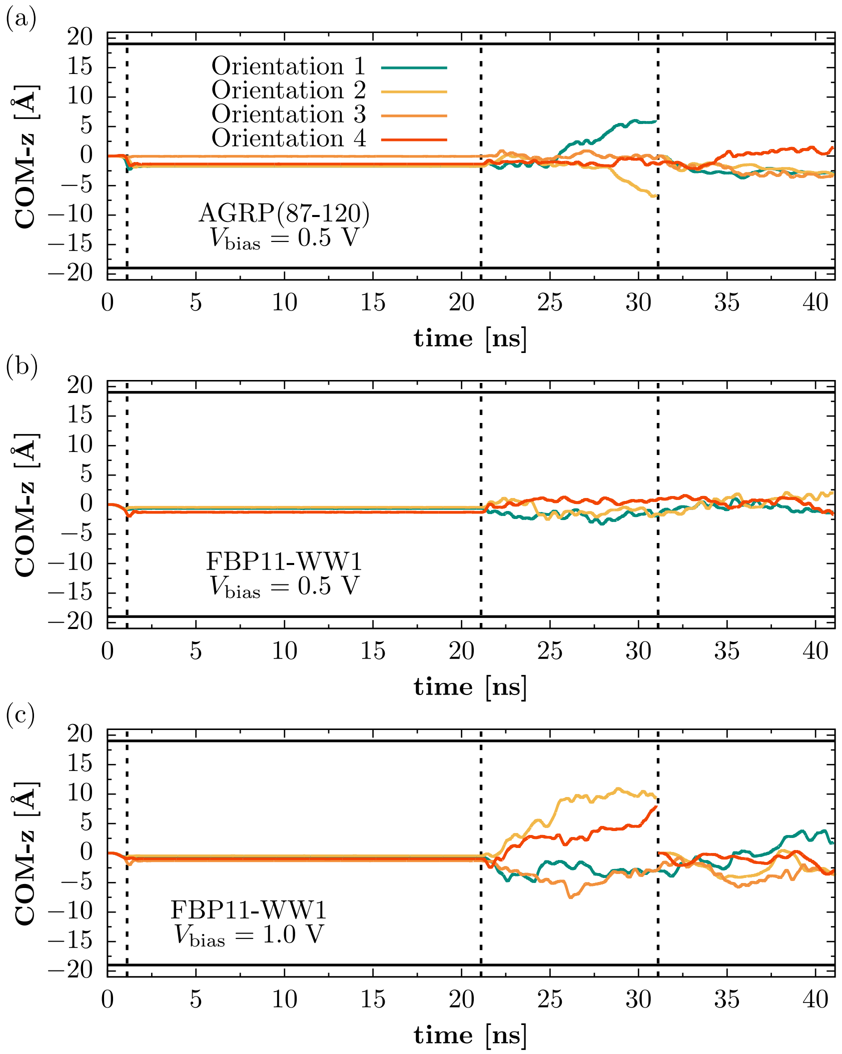

Figure 1: COM z-direction for miniproteins as a function of the simulation time. The results have been smoothed out with a LPF using a 1 GHz cutoff frequency.

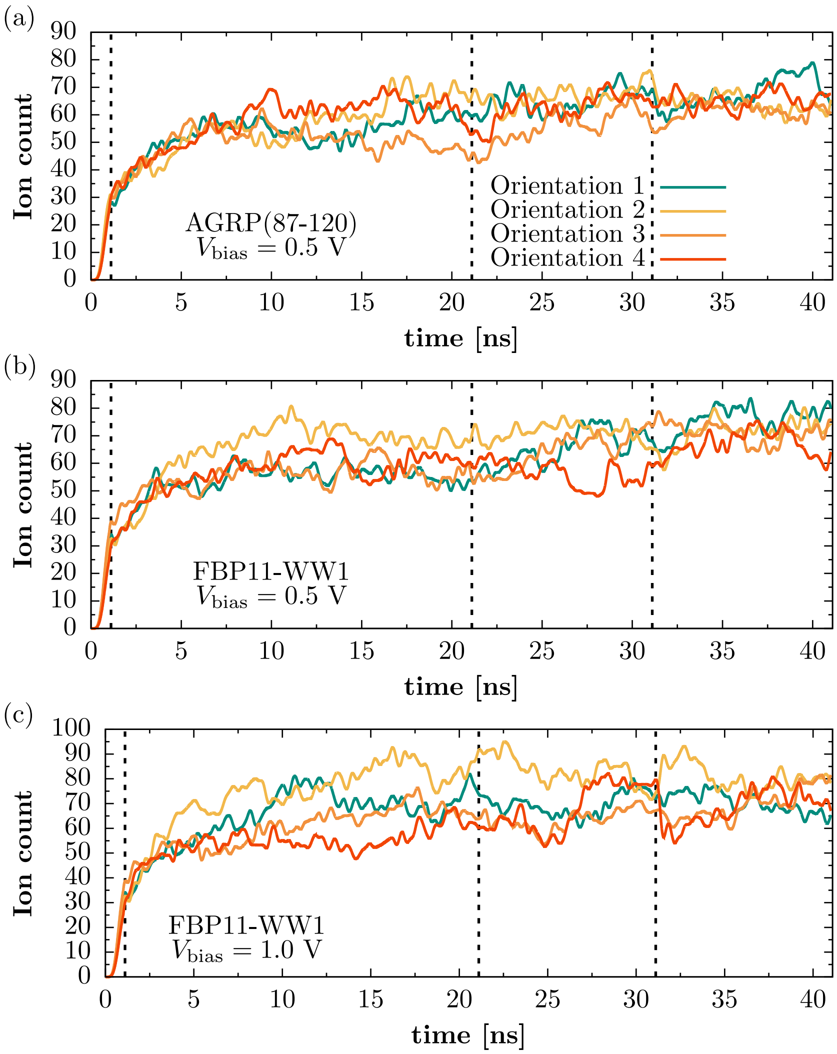

Figure 2: The instantaneous number of ions inside the occupied pore as a function of simulation time. The results have been smoothed out with a LPF using a 1 GHz cutoff frequency.

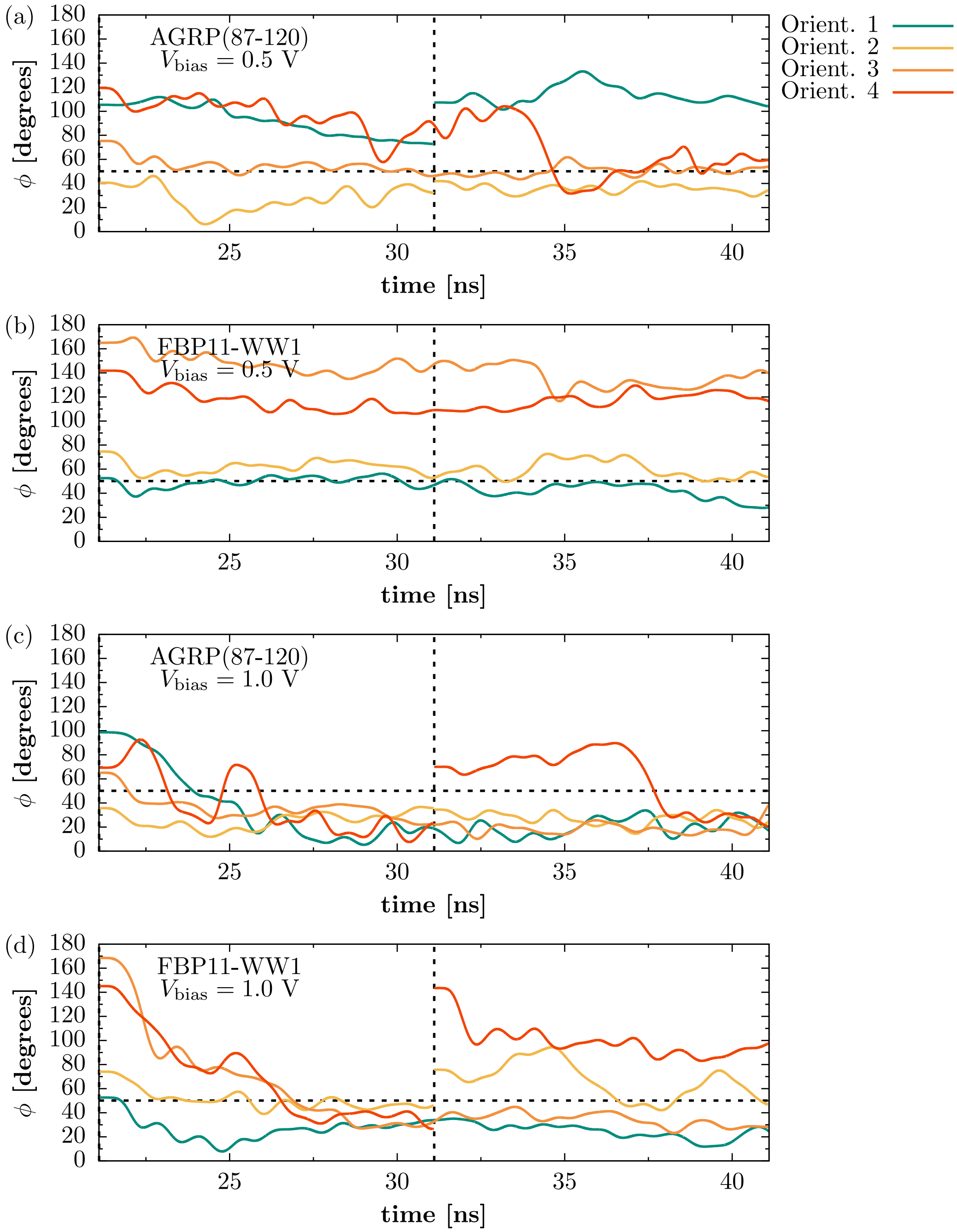

Figure 3: Miniprotein dipole moment direction as a function of time. The dipole direction was determined by the angle ϕ made between the dipole vector and the z-axis along the positive direction. Full alignment with the external field happens at 0° and anti-alignment happens at 180°.

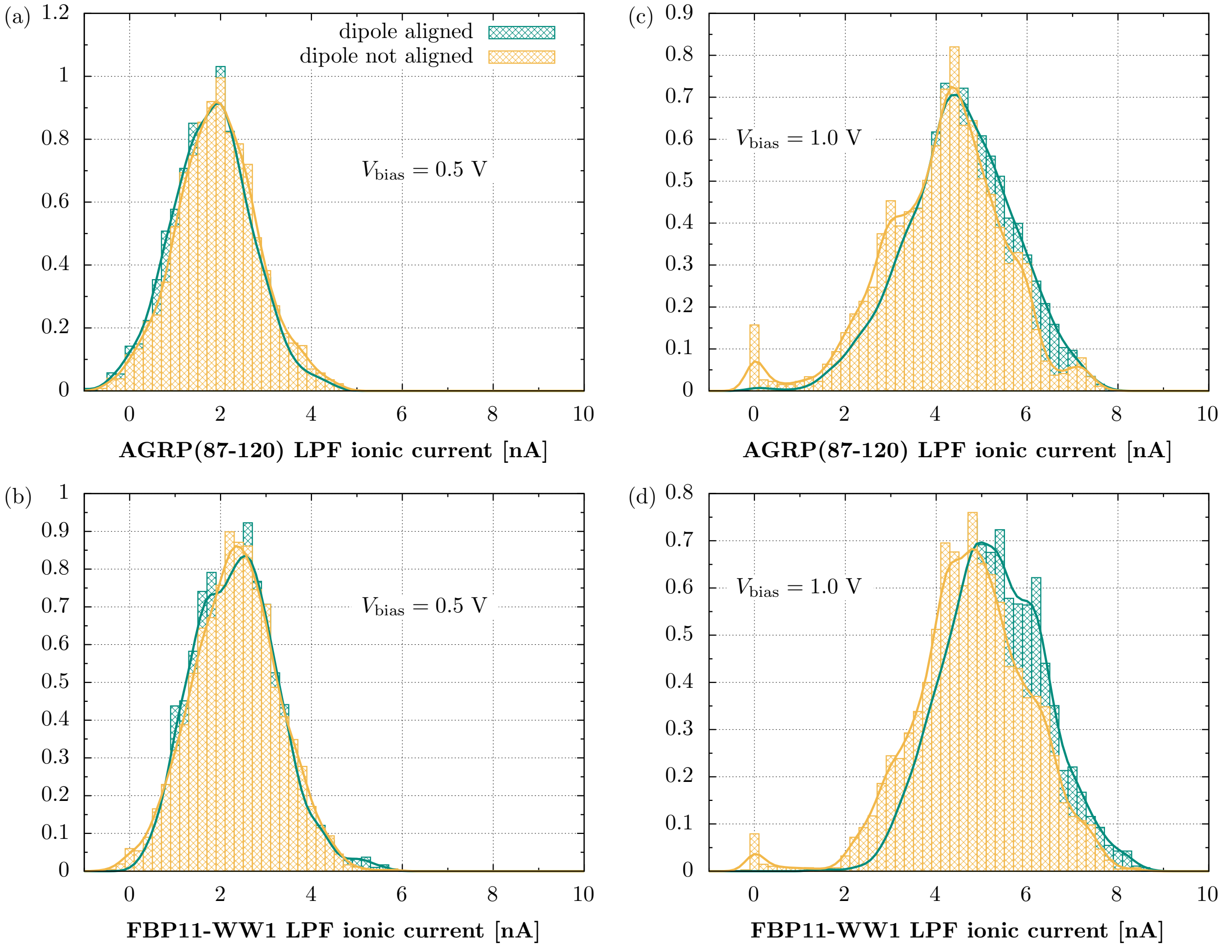

Figure 4: Comparing LPF ionic current histograms for when miniproteins are aligned or not aligned with the external field. To compare these two scenarios, histograms are normalized to the number of data points available for each distribution.

Miniprotein information

Information about the two miniproteins investigated is shown in table 1. Identity is a property that measures the number of amino acids that, excluding gaps, exactly match in both sequences. Figure 5 shows the optimal alignment of the two miniprotein sequences, where we find an identity value of 17.39 %. The identity indicates similarities in the primary structure of the two miniproteins and shows that their sequence differs significantly.

```
AGRP(87-120):   CVRLHESCLGQQVPCCDPAATCYCRFFNAFC----YCR---
FBP11-WW1:      ---------GSWTEHKSPDGRTY--YYNTETKQSTWEKPDD
```

Figure 5: Aligned miniprotein sequences, with amino acids represented by their single-letter residue codes and gaps by a dash symbol. Identity value and the aligned sequence were obtained with Clustal Omega[1].

Table 1: Selected miniproteins. Their structure has been determined by NMR spectroscopy in an aqueous environment, and their files were obtained from the Protein Data Bank (PDB)[2].

PDB	name	length[a] (Å)	mass (Da)	volume[b] (Å^3)	residues
1ZR7	FBP11 WW1	30.7	3587.7	8.47×10^3	30
1MR0	AGRP(87-120)	31.6	3884.5	8.90×10^3	34

[a] Largest distance between any two atomic centers; [b] Approximate volume determined using MDTools *MolVolume* with a probe radius of 2.0 Å and grid size of 0.1 Å.

Relative permittivity silicon nitride

To compute the permittivity we ran molecular dynamics simulations on a silicon nitride sphere. The sphere had a radius of 36.5 Å and was placed in vacuum inside a simulation cube 180 Å in length and held at a constant temperature of 298 K using a Lowe-Andersen thermostat. The relative permittivity ϵ_r was computed from

$$E_z = \frac{3}{2+\epsilon_\mathrm{r}} E_\mathrm{ext} \ ,$$

where E_{ext} is the field produced by the bias voltage applied along the z-direction and E_z is the field inside the sphere. We assumed the sphere had a uniform charge density such that the electric field inside depended only on its surface charge density. Inside the sphere, the field was computed using

$$E_z = -\frac{1}{3\epsilon_0} P_z \ ,$$

where P_z is the polarization and ϵ_0 is the permittivity of free space. The polarization was computed from the time-averaged dipole moment change and time-averaged volume using

$$P_z = \frac{\langle \Delta D_z \rangle_t}{\langle V \rangle_t} \ .$$

We explored ϵ_r at different harmonic restraints and bias voltages, with results shown in figure 6.

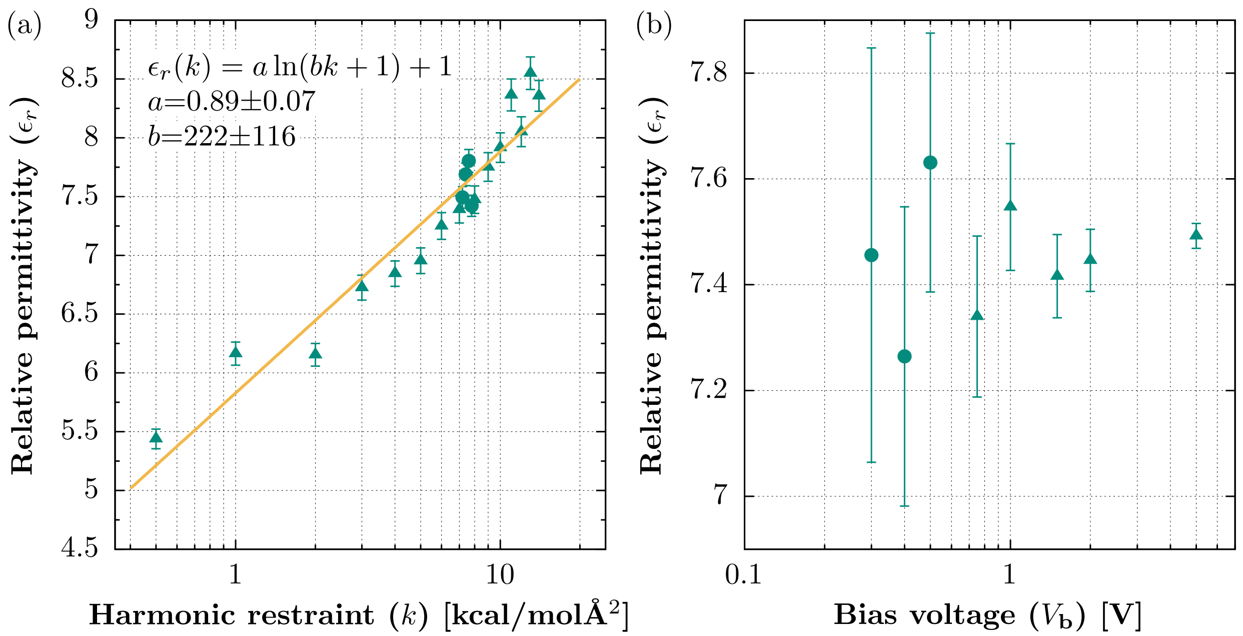

Figure 6: (a) Relative permittivity as a function of harmonic restraints. Triangles represent values obtained from 1.0 ns-long simulations and circles from 1.6 ns-long simulations. We fitted the results to a logarithmic weighted best fit. (b) Relative permittivity for different applied bias voltages. Values represented by triangles and circles were obtained from 1 ns and 2 ns-long simulations, respectively. Uncertainties in (a) and (b) are derived from fluctuations in the computed time-averaged dipole moment and sphere's volume.

Pore surface silicon-nitrogen ratio

All atoms in the silicon nitride membrane, except those near the pore surface, were given a harmonic restraint of 1 kcal/(molÅ^2). The system was placed in vacuum, minimized for 0.01 ns, equilibrated for 0.15 ns at 298 K using a Lowe Andersen thermostat, and again minimized for 0.01 ns. Surface silicon and nitrogen counts were based on trajectory files stored 0.1 ps, with results shown in figure 7.

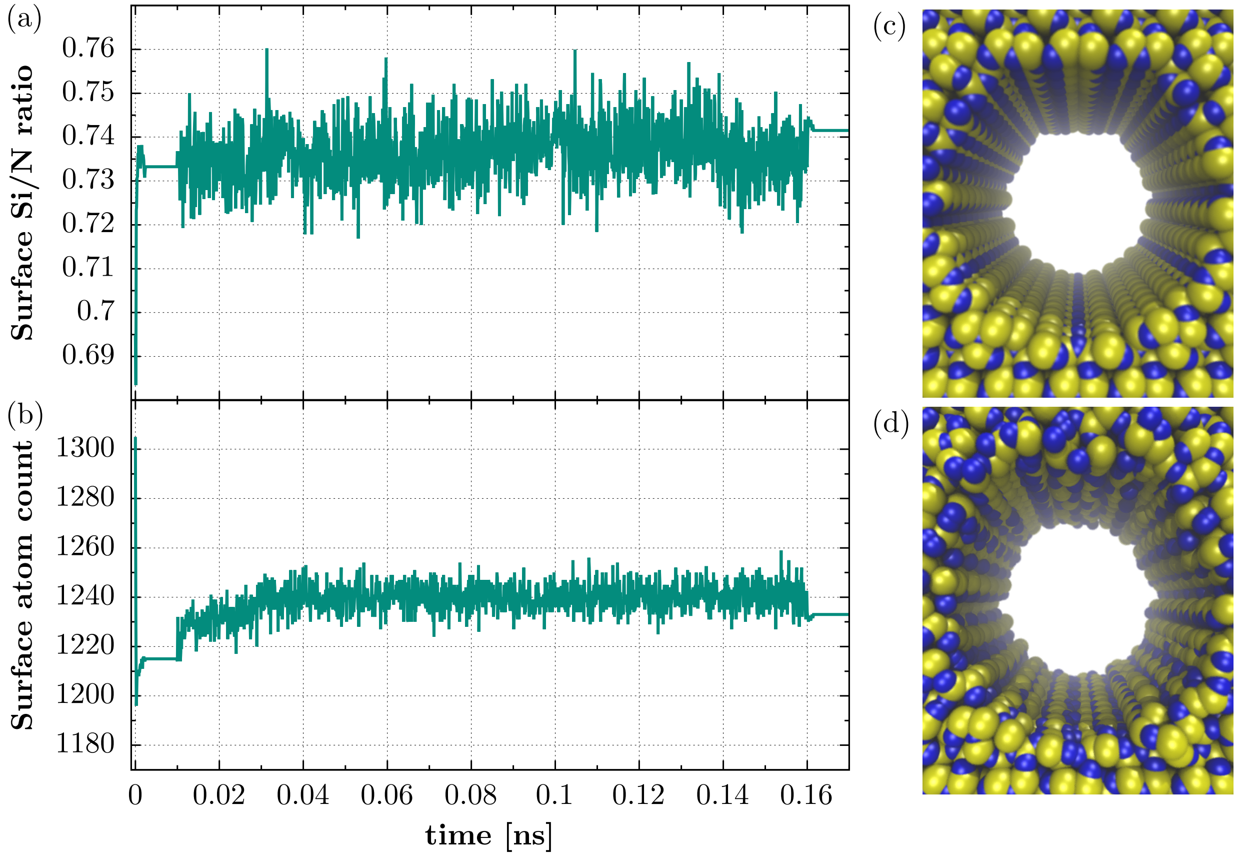

Figure 7: (a) Silicon-nitrogen atom ratio at the surface of the nanopore. (b) The number of atoms being considered as surface atoms in the calculation of the silicon/nitrogen ratio. (c)(d) Before and after snapshots of the pore surface atomic arrangement.

Ion conductivity

The Lowe-Andersen thermostat keeps a target temperature by modifying the velocity of pairs of particles within a search radius R_T, where each pair has a probability $\Gamma\,\Delta t$ of colliding

with the heat bath[3,4]. Thermostat applies a shear stress that contributes with an artificial viscosity

$$\eta^T \propto \frac{\pi \rho^2 R_T^5 \Gamma}{75\, m} ,$$

where m and ρ in our case are the mass and density of water, Γ is the collision rate and R_T the search radius[3,4]. To adjust the ion conductivity in MD simulations, we benefited from η^T. In principle, parameters R_T and Γ can be modified, but it is desirable to have a large collision rate that keeps the temperature constant and achieves a high rate of diffusion[3].

We based our ion conductivity calibration on methods presented in Aksimentiev and Schulten[5]. The reference molar conductivity $\kappa = 14.985$ S/m for potassium chloride was determined from textbook ionic molar conductivities in bulk solution[6]. We simulated a cube of water 4 nm in length with a 1 M concentration of KCl using target temperature 298 K. For a fixed R_T value and after a 0.05 ns equilibration (NPT conditions with 1 atm), we simulated the system for 1.0 ns (NVT conditions) under a range of 0.1–5.0 V bias voltages. Results from the simulations are shown in figure 8.

Energy convergence

We were interested in mesh cutoff and cell dimension parameters that gave a well-converged ground state energy for silicon nitride and amino acid in vacuum. We examined these parameters for two systems: (1) amino acid glutamine with 20 atoms in total and (2) silicon nitride together with glutamine with 300 atoms in total. We employed a double-ζ polarized basis set with the van der Waals functional DRSLL. Convergence was achieved using Pulay with a mixing factor of 0.1 for system (1) and 0.02 for system (2), and a history of 5 self-consistent cycles. The convergence results are shown in figure 9.

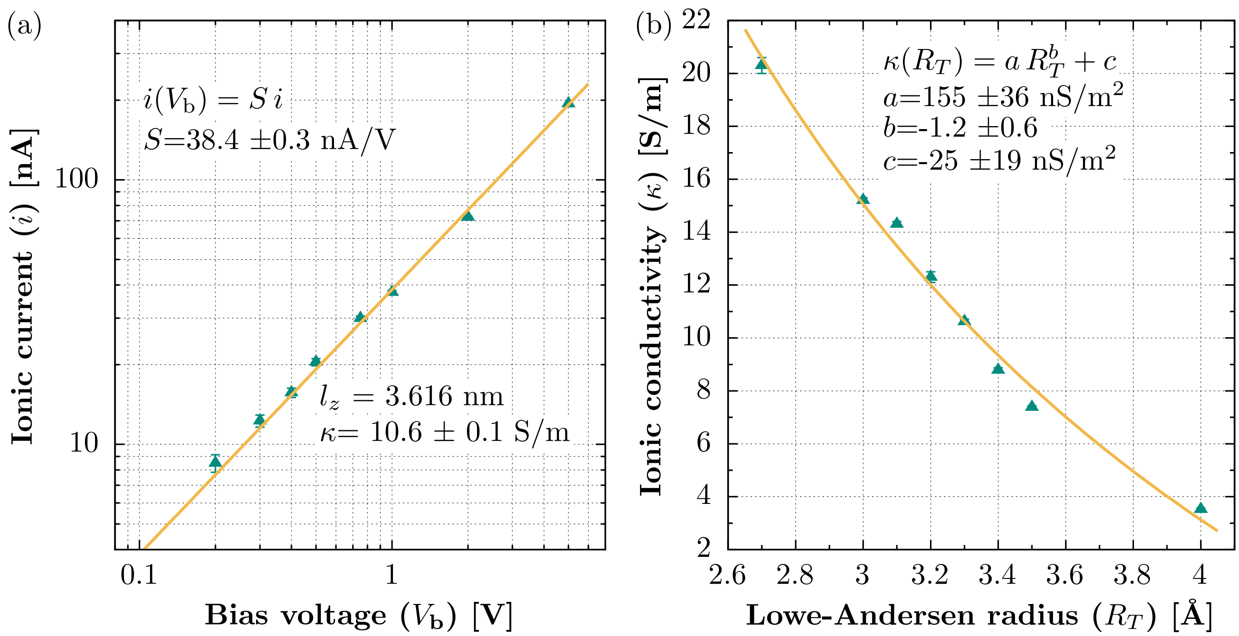

Figure 8: (a) Ionic current as a function of applied bias voltage for a fixed Lowe-Andersen radius $R_T = 3.0$ Å. The ionic current was computed over the entire length of the simulation box, using ions' trajectories stored every 0.1 ps. The slope of the best fit is the conductance S. (b) Ion conductivity for a range of R_T values, based on the slope of the weighted linear best fit of the current vs. bias voltage plots.

Terminal velocity

For the dwell time, we determined the constant A based on the protein translocation without interaction with the pore surface. We imagined the protein experiences some acceleration subject to the electric field F_e and drag force F_d

$$m\,a = F_\mathrm{e} - F_d \; . \tag{1}$$

From MD simulations, we estimated that the leading contribution from the drag force happens in the limit of small velocity ($\mathcal{O}(0.1$ m/s) for $\Delta d = 2$ nm, $t = 10$ ns). The miniprotein moves through a viscous fluid and the drag force is proportional to the velocity. This effect is shown in figure 10. The net force can be written as

$$\frac{dv}{dt} = a_e - k_d\,v \; , \tag{2}$$

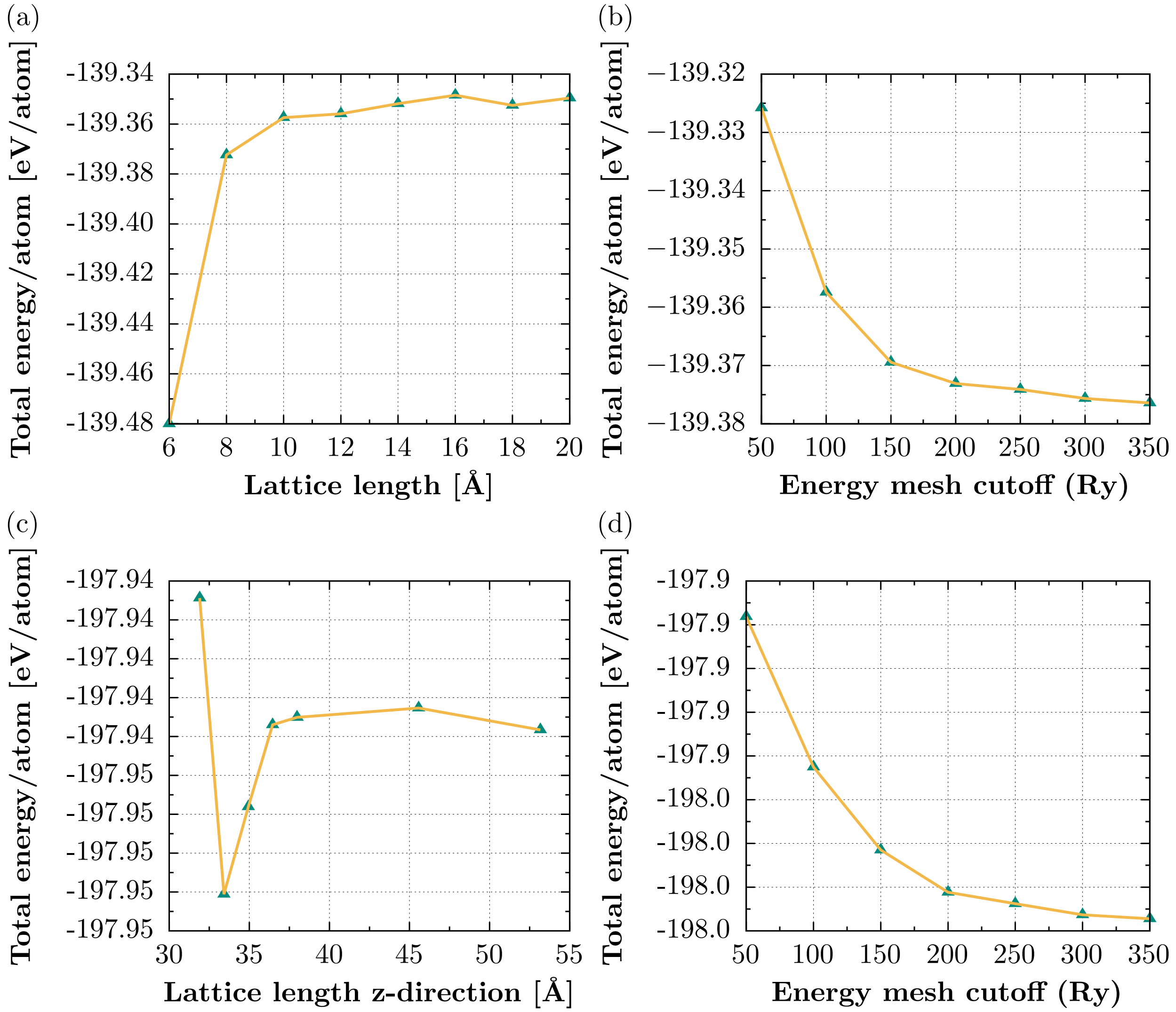

Figure 9: (a) Ground state energy for ASN as a function of the separation distance between mirror images (all directions). (b) Ground state energy for ASN for increasing mesh cutoff energy values. (c) Ground state energy for silicon nitride slab and ASN system as a function of separation distance along the z-direction between mirror images. (b) Ground state energy for silicon nitride slab and ASN for increasing mesh cutoff energy values.

where $a_d = V\,q/d\,m$ and $k_d = 6\,\pi\,\nu\,r\,/m$. Here we assumed the miniprotein was a sphere of mass m, radius r (taken to be half the length of the protein), and $\eta_{\text{water}(25^o)} = 0.89$ mPa·s is the viscosity of the medium. We integrated this differential equation to obtain the velocity and position of the protein as a function of time. We found the miniprotein experiences a short acceleration period and quickly reaches terminal velocity within the first few ps, as shown in figure 10(b). From the position as a function of time

$$x(t) = \frac{a_e}{k_d} t + \frac{a_e}{k_d^2}\left(e^{-k_d\,t} - 1\right)\ , \tag{3}$$

we numerically solved the dwell time based on the thickness of the membrane as shown in figure 10(c).

References

1. Sievers, F.; Wilm, A.; Dineen, D.; Gibson, T. J.; Karplus, K.; Li, W.; Lopez, R.; McWilliam, H.; Remmert, M.; Söding, J.; Thompson, J. D.; Higgins, D. G. Fast, scalable generation of high-quality protein multiple sequence alignments using Clustal Omega. *Molecular Systems Biology* **2011**, *7*, 539, Publisher: John Wiley & Sons, Ltd.

2. Berman, H.; Henrick, K.; Nakamura, H. Announcing the worldwide Protein Data Bank. *Nature Structural & Molecular Biology* **2003**, *10*, 980–980.

3. Koopman, E. A.; Lowe, C. P. Advantages of a Lowe-Andersen thermostat in molecular dynamics simulations. *The Journal of Chemical Physics* **2006**, *124*, 204103, _eprint: https://doi.org/10.1063/1.2198824.

4. Lowe, C. P. An alternative approach to dissipative particle dynamics. *Europhysics Letters (EPL)* **1999**, *47*, 145–151, Publisher: IOP Publishing.

5. Aksimentiev, A.; Schulten, K. Imaging α-Hemolysin with Molecular Dynamics: Ionic

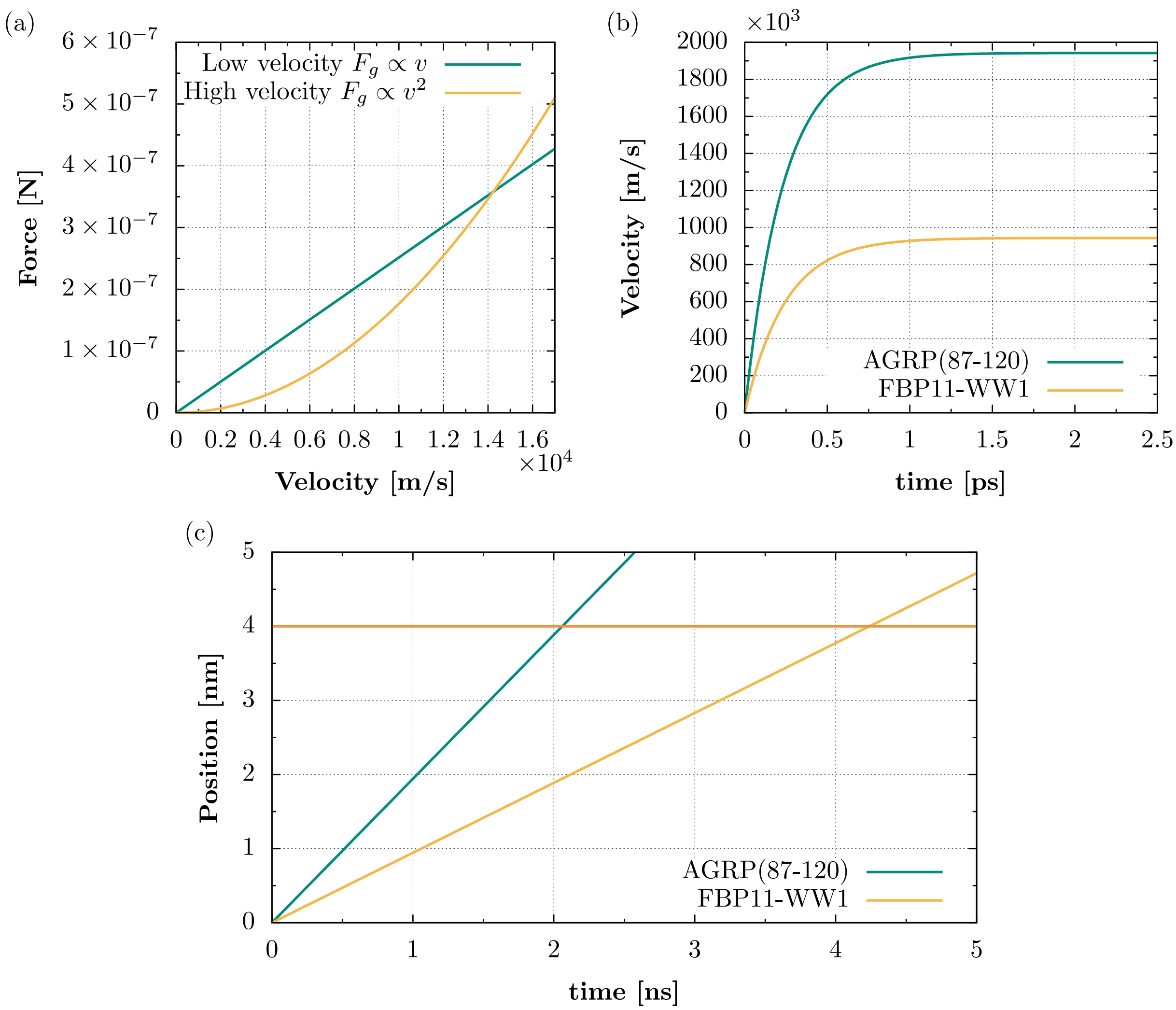

Figure 10: Determining the dwell time constant A for each miniprotein based on the electric field and drag forces. (a) Approximation of the drag force from two limiting cases for large and small velocity. (b) Miniproteins reach terminal velocity within the first few ps. (c) Calculation of A based on the movement of the miniproteins over time and approximate thickness of the silicon nitride membrane.

Conductance, Osmotic Permeability, and the Electrostatic Potential Map. *Biophysical Journal* **2005**, *88*, 3745–3761, Publisher: Elsevier.

6. Coury, L. Conductance Measurements Part 1 : Theory. *Current Separations* **1999**, *18*, 91–96.
